# Nearly coming full circle: The 13th ISABS–Mayo Clinic Conference, Split, Croatia, June 17–20, 2024

**DOI:** 10.3325/cmj.2024.65.167

**Published:** 2024-06

**Authors:** Stanimir Vuk-Pavlović, Dragan Primorac

**Affiliations:** International Society for Applied Biological Sciences, Zagreb, Croatia; dragan.primorac@svkatarina.hr; *vuk@mayo.edu*

Twenty-seven years after the first European-American Intensive Course in PCR-Based Clinical and Forensic Testing, the 13th ISABS–Mayo Clinic Conference is returning to Split, its original venue. This homecoming symbolizes that the conference series is fulfilling the promise implicitly made at its inception.

**Figure 1:**
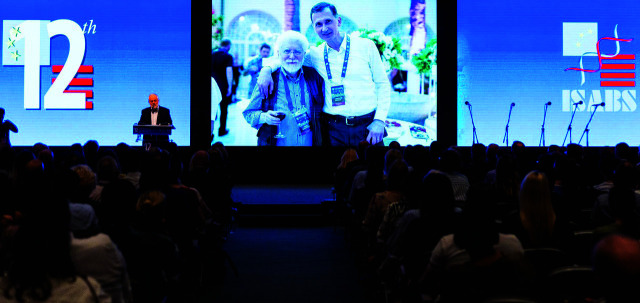
At the 12th Conference in 2022, Prof. Stanimir Vuk-Pavlović eulogizes Moses Samuel Schanfield (1944-2021), the pioneer of ISABS Conferences. The image on the screen shows Profs. Schanfield (left) and Dragan Primorac.

In 1997, the inaugural conference arose from the need to introduce to the region the then-novel applications of polymerase chain reaction in forensic medicine. This need was not purely academic but was sadly motivated by the necessity of identifying victims of the Yugoslav dissolution war (1991-1995) exhumed from mass graves (1). The central figure of this event was Moses Schanfield, a pioneer in DNA analysis for forensic medicine (Figure 1). The conference revolved around a course on PCR applications in both forensics and clinical diagnostics. The focus on application and education has informed subsequent conferences, and their main producer, the International Society for Applied Biology (ISABS), ever since.

The program's method-based and application-oriented focus resulted early in the inclusion of anthropological genetics. Subsequent biennial conferences have continued to present cutting-edge advances in methods and bring together leading experts in the field. The integration of forensic genetics with anthropological genetics became a defining feature of these conferences. Regular participation by top forensic scientists has contributed to establishing a forensic master-of-science program at the University of Split, which has been successfully educating new generations since 2009. These conferences were also influenced by the research on deciphering Neanderthal genomes involving remains from Vindija Cave in Croatia and the collaboration with Croatian scientists who assisted the Nobel Prize-winning work by Svante Pääbo. Thus, both the forensic and anthropological tracks of this conference series have nearly come full circle.

**Figure 2:**
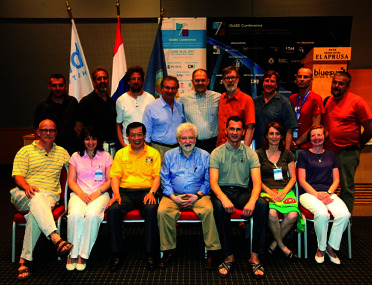
Scientists from reputable international institutions contributed to DNA identification of war victims in Croatia, Slovenia, and Bosnia and Herzegovina. At the 7th ISABS Conference, 2011, most of these scientific achievements, especially those related to DNA identification methods, were exclusively presented for the first time.

Since its third iteration in 2003, Mayo Clinic has partnered with ISABS, contributing to medically oriented programs that have naturally emphasized different topics across conferences (Figure 2). This 13th Conference highlights new developments, such as regenerative medicine, immunotherapy, artificial intelligence/machine learning, and senescence research, among others. These areas are expected to continue expanding at future conferences to meet educational needs and technology transfer within the region. Additionally, the growing emphasis on regenerative medicine has led organizations with aligned interests to host satellite symposia alongside or before the conferences. This year, the International Regenerative Medicine Experts Society (IARMES), the European Olympic Committee’s Medical Commission, the 2nd Croatian Personalized (Precision) Medicine Conference, and 9th Croatian Human Genetics Conference are set to conduct such symposia.

The tradition of Nobel laureates delivering key lectures at conferences began with the 6th Conference in 2009. This year’s panel features Sir Richard Roberts (Physiology or Medicine, 1993), Gregg Semenza (Physiology or Medicine, 2019), Svante Pääbo (Physiology or Medicine, 2022), and Aaron Ciechanover (Chemistry, 2004). They will contribute to the “Nobel Spirit,” the unique and distinguished panel exploring the impact of science in the contemporary world, which will be covered by national broadcasting media.

This year, ISABS will celebrate its activities by showcasing four recently published books that reflect its work or have its support. Henry A. Erlich’s Genetic Reconstruction of the Past: DNA Analysis in Forensics and Human Evolution (2), a book by a seasoned ISABS Conferences speaker and PCR development pioneer, chronicles the concurrent progress in forensic science and molecular anthropology. In Gunshot in Croatia, Xiaping Jiang recounts the international forensic efforts related to the 1991-1995 war (3). Additionally, ISABS has supported two multi-author volumes: Forensic DNA Applications: An Interdisciplinary Perspective, 2nd Edition (4) edited by Dragan Primorac and Moses Schanfield, and Pharmacogenomics in Clinical Practice (5) edited by Dragan Primorac, Wolfgang Höppner, and Lidija Bach-Rojecky.

Throughout its history, ISABS has found a consistent partner in the *Croatian Medical Journal* (CMJ). Both emerged from the Croatian Homeland War (1991-1995): ISABS for the reasons explained above and *CMJ* for its aim to share the unique experience of Croatian war medicine globally. Consequently, the *CMJ* has become the ISABS' official journal thanks to the continuous dedication and tireless efforts of the *CMJ* editorial teams. The collaboration has flourished over time, yielding more than 100 publications, some of which rank among the most cited in the *CMJ*’s history. Our partnership culminates in the current thematic issue devoted to the 13th ISABS-Mayo Clinic conference and presents the highest number of conference papers to date.
